# Minimizing resource overheads for fault-tolerant preparation of encoded states of the Steane code

**DOI:** 10.1038/srep19578

**Published:** 2016-01-27

**Authors:** Hayato Goto

**Affiliations:** 1 Frontier Research Laboratory, Corporate Research & Development Center, Toshiba Corporation, 1, Komukai Toshiba-cho, Saiwai-ku, Kawasaki-shi, 212-8582, Japan

## Abstract

The seven-qubit quantum error-correcting code originally proposed by Steane is one of the best known quantum codes. The Steane code has a desirable property that most basic operations can be performed easily in a fault-tolerant manner. A major obstacle to fault-tolerant quantum computation with the Steane code is fault-tolerant preparation of encoded states, which requires large computational resources. Here we propose efficient state preparation methods for zero and magic states encoded with the Steane code, where the zero state is one of the computational basis states and the magic state allows us to achieve universality in fault-tolerant quantum computation. The methods minimize resource overheads for the fault-tolerant state preparation, and therefore reduce necessary resources for quantum computation with the Steane code. Thus, the present results will open a new possibility for efficient fault-tolerant quantum computation.

Quantum computers are expected to outperform conventional classical computers for various problems^1^, such as factoring of large integers^2^ or machine learning problems^3^,^4^. However, the realization of quantum computers is extremely difficult because of decoherence (destruction of quantum coherence), which makes quantum bits (qubits) much more fragile than classical bits. A standard approach to this problem is to use quantum error-correcting codes for fault tolerance^1^,^5^,^6^,^7^,^8^,^9^. By using such codes, long quantum computation can be performed reliably as long as physical error probabilities are sufficiently low.

Steane’s seven-qubit code^10^ is one of the best known quantum codes. (The definition and basic properties of the Steane code are given in Methods.) This code has a good property that the so-called Clifford gates and the computational basis measurement can be implemented transversally^1^ (in a bitwise manner), where the transversal implementation guarantees fault tolerance automatically. In addition to these basic operations, we need fault-tolerant preparation of the following two types of state for universal quantum computation: a computational basis state, 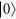 or 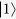, and a nonstabilizer state, such as 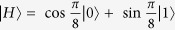 or 
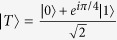
. The nonstabilizer states allow one to perform non-Clifford gates together with the Clifford operations. In this paper, we refer to the nonstabilizer states as the magic states, which were named after magic state distillation^11^. Unfortunately, the fault-tolerant preparation of these states encoded with the Steane code requires complicated processes and large resource overheads^1^,^6^,^12^,^13^,^14^.

To minimize the resource overheads for the fault-tolerant state preparation, here we propose new state preparation methods for the Steane code. To prepare the zero state 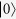 and the magic state 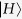 encoded with the Steane code in a fault-tolerant manner, we use one and eighteen physical ancilla qubits, respectively. These are significantly fewer than those for conventional methods. Thus, the present methods will open a new possibility for efficient fault-tolerant quantum computation.

## Results

### Preparation of encoded zero state

**Figure 1:**
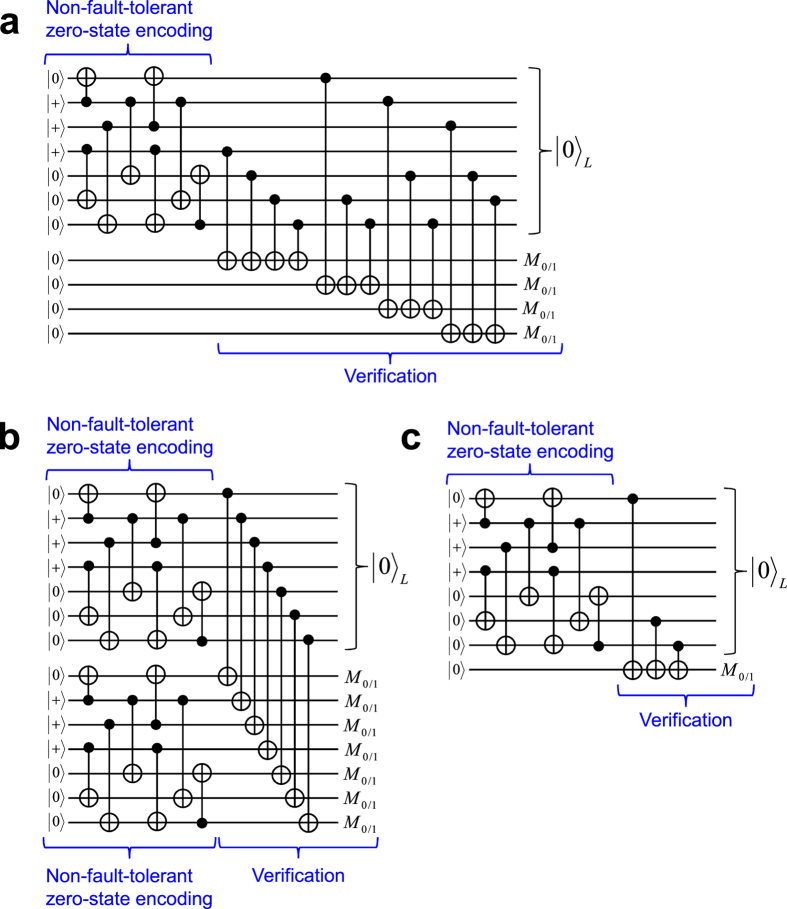
Quantum circuits for fault-tolerant preparation of 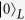 encoded with the Steane code. (**a**) Conventional method with four physical ancilla qubits for verification. (**b**) Conventional method with a logical ancilla qubit in 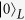 for verification. (**c**) Proposed method with one physical ancilla qubit for verification. Here  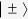  is defined as  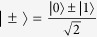 and *M*_0/1_ denotes the computational basis measurement of a physical qubit.

There are two conventional methods for fault-tolerant preparation of the logical zero state, 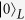, encoded with the Steane code, which are shown in Fig. 1a,b. Both consist of two parts: non-fault-tolerant encoding and verification.

For the non-fault-tolerant encoding of 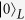, we use the method proposed by Paetznick and Reichardt^15^. This method allows us to prepare 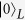 only with eight controlled-NOT (CNOT) gates, unlike the conventional one^12^,^13^,^14^, which requires nine CNOT gates.

The verification in Fig. 1a is based on the measurements of four stabilizer operators of 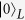 (*IIIZZZZ*, *ZIIIIZZ*, *IZIIZIZ*, *IIZIZZI*) with four physical ancilla qubits. (In this paper, the identity and Pauli operators are denoted by *I*, *X*, *Y*, and *Z*.) On the other hand, the verification in Fig. 1b do the same measurements of the stabilizer operators *at once* by using a logical ancilla qubit in 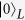. In both cases, the whole process is repeated until no error is detected in the verification. This postselection guarantees the fault tolerance of the output state. (In this paper, the “fault tolerance” is defined such that logical error probabilities are of the second order of physical error probabilities, because the Steane code can correct single-qubit errors. See Methods.)

Here it should be noted that in the above two verification methods, only *Z* stabilizer operators are measured to detect *X* errors, and *X* stabilizer operators are not. This is because no *Z* errors are harmful for 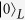 encoded with the Steane code. This fact comes from *error degeneracy*, which is a special property of quantum codes that classical codes do not possess. The error degeneracy is also a key to understanding the present method described below. Because of the *Z* stabilizer operators of 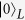 given above, arbitrary *Z* errors are equivalent to no error or single-qubit *Z* errors. This equivalence is the error degeneracy. The single-qubit *Z* errors can be corrected by the Steane code. Thus, the fault-tolerance is achieved without measuring *X* stabilizer operators.

The method proposed in this paper is shown in Fig. 1c. This method also consists of non-fault-tolerant encoding and verification. In this method, we use only *one* physical ancilla qubit for the verification. Thus, the present method minimizes the resource overhead for the fault-tolerant preparation of 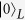.

The principle of the present verification method is explained as follows. Here we assume that at most one physical error occurs, because fault tolerance is achieved by eliminating such errors by the definition mentioned above.

After the first six CNOT gates in the encoding in Fig. 1c, the seven-qubit state without the ancilla qubit is described by the following stabilizer operators: *ZZZIIII*, *XXIIXII*, *XIXIIIX*, *IIIXIXX*, *IZIIZII*, *IIIZIZI*, and *IIZZIIZ*. Because of these, arbitrary two-qubit errors due to the six CNOT gates are equivalent to single-qubit errors. Thus, two-qubit errors in the encoding are induced only by the last two CNOT gates. The two-qubit *X* errors can be detected and eliminated by the measurement of *ZIIIIZZ* with the ancilla qubit. (Note that *ZIIIIZZ* is one of the stabilizer operators of 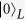.) On the other hand, error propagation via the ancilla qubit induces only correlated *Z* errors, not *X* errors, which are not harmful for 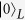 as mentioned above. Thus, the simple method shown in Fig. 1c can achieve fault tolerance.

To evaluate the performance of the present method qualitatively, we performed numerical simulation of a logical CNOT gate, which consists of transversal physical CNOT gates followed by error-correcting teleportations with logical Bell states^7^,^16^. The reason why we examine not a logical zero state preparation but a logical CNOT gate is as follows. First, if we measure a prepared logical zero state in the computational basis to evaluate this, then we obtain only the information on *X* errors and miss that on *Z* errors on the zero state. Note that the *Z* errors are not negligible because they affect the error probabilities of, e.g., a logical CNOT gate. (Four logical zero states are consumed for two logical Bell states used for two error-correcting teleportations in the logical CNOT gate.) The effect of the *Z* errors can be examined by evaluating the logical CNOT gate. Second, if a prepared logical zero state has a logical error, then the logical CNOT gate with error-correcting teleportations using the zero state has also a logical error. That is, it is sufficient to examine a logical CNOT gate to ensure the fault tolerance of a prepared logical zero state.

In the present simulation, we used an error model such that physical two-qubit gates are imperfect with error probability *p*_*e*_ and the other physical operations (state preparations, one-qubit gates, and qubit measurements) are perfect^16^,^17^. This simple model is valid as follows. First, two-qubit gates have higher error probabilities than the other physical operations for most physical systems. Second, they are used most frequently in fault-tolerant quantum computation^6^. Finally, the other physical operations are always followed or preceded by two-qubit gates, and therefore we can assume that their error probabilities are included in those of two-qubit gates. Note that this assumption does not affect the fault tolerance. That is, if the fault tolerance holds in the present model, then this is the case in the model including other errors. (Including other errors will result in the increase of logical error probabilities by some factor.) The model for an imperfect two-qubit gate is the standard one^7^,^16^,^17^: one of the 15 two-qubit Pauli errors occurs on the control and target qubits with probability *p*_*e*_/15 after a perfect gate operation. The simulator used here is the stabilizer simulator based on ref. 18.

**Figure 2:**
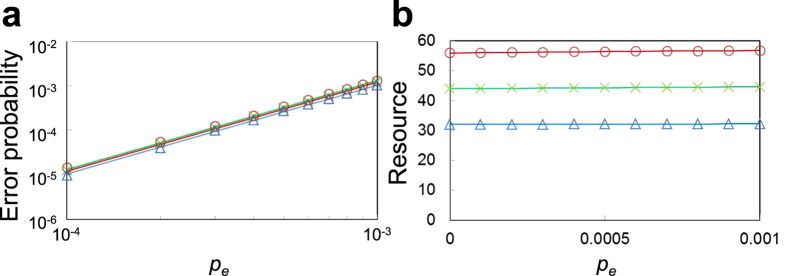
Simulation results of a logical CNOT gate. (**a**) Log-log plot of error probabilities of a logical CNOT gate. The straight lines are the fits of 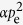 to the plots, where *α* is a fitting parameter. (**b**) Linear plot of resources of a logical CNOT gate, where the resource is defined as the total number of physical qubits. In both (**a**) and (**b**), the crosses, the circles, and the triangles correspond to the cases where logical zero states are prepared by the methods shown in Fig. 1a–c, respectively. *p*_*e*_ denotes the error probability per physical two-qubit gate.

From the simulation results, we estimated the error probability and total number of physical qubits for a logical CNOT gate, which are shown in Fig. 2a,b, respectively. In Fig. 2, the crosses, the circles, and the triangles correspond to the cases where logical zero states, which are used for logical Bell states for error-correcting teleportation, are prepared by the methods shown in Fig. 1a–c, respectively. (See Methods for the details of the simulation.)

### Preparation of encoded magic state

A recent standard approach to fault-tolerant preparation of encoded magic states is based on magic state distillation^11^,^19^,^20^,^21^,^22^,^23^. However, the distillation approach requires at least five logical magic states encoded in a non-fault-tolerant manner and eight logical two-qubit gates^23^, and therefore the resource requirements are high. Although a detailed study on the distillation approach is beyond the scope of the present work, the present method will achieve fewer resource requirements than the distillation approach, because the present method requires only 25 physical qubits for fault-tolerant magic-state preparation, as explained below. (On the other hand, the distillation approach may achieve a lower logical error probability by some factor.)

**Figure 3:**
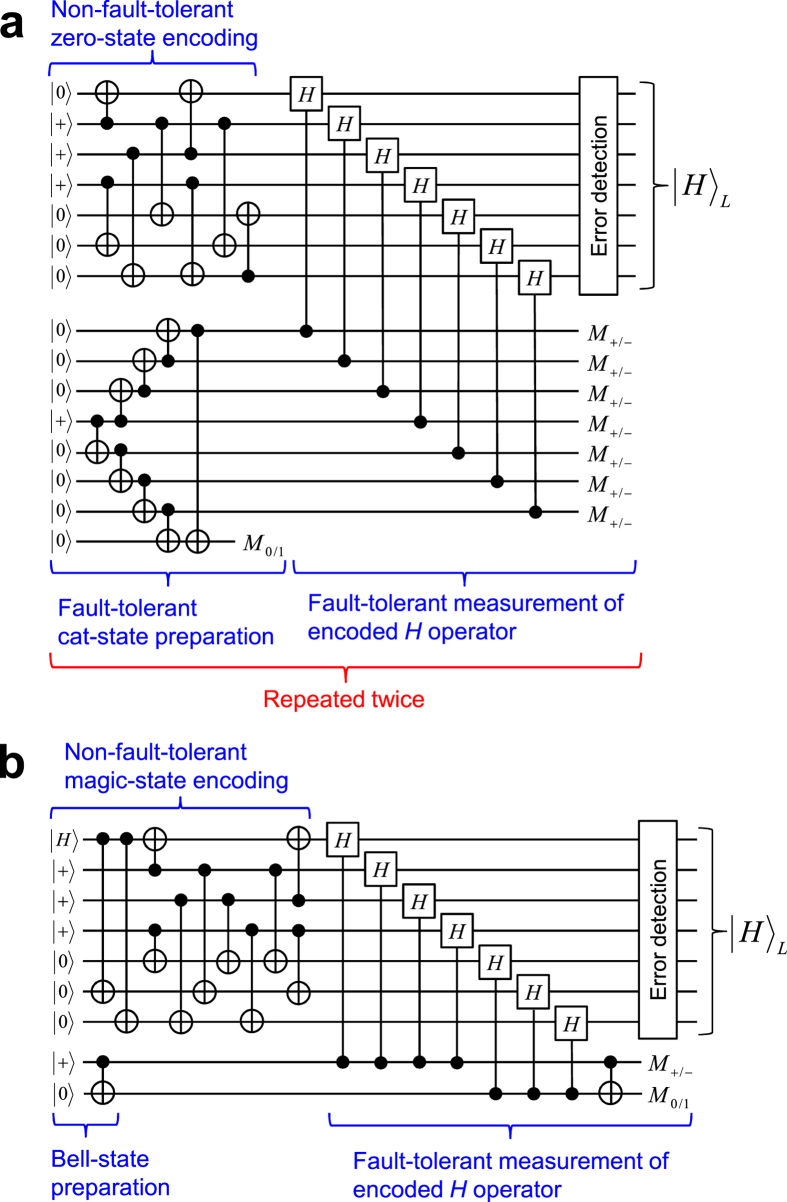
Quantum circuits for fault-tolerant preparation of  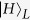 encoded with the Steane code. (**a**) Conventional method. The whole procedure without the preparation of the logical zero state is repeated twice. (**b**) Proposed method. In both (**a**) and (**b**), the error detection with the Steane code is implemented by, e.g., error-detecting teleportation^7^,^16^.

**Figure 4:**
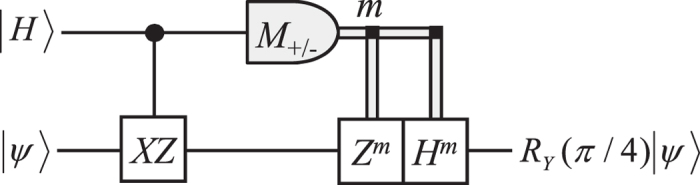
Quantum circuit for a non-Clifford gate *R*_*Y*_(*π*/4) with a magic state 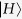. *M*_+/−_ denotes the {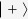, 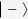} basis measurement and *m* denotes the measurement result, where *m* equals 0 and 1 for the results  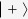 and  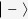, respectively.

On the other hand, for the Steane code, there is a more conventional method based on fault-tolerant measurement^1^,^14^, which is shown in Fig. 3a. In this case, a magic state is 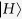, which is the eigenstate of the Hadamard operator, *H*, with the eigenvalue of +1. We can perform a non-Clifford gate 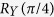 with 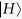 and Clifford operations, as shown in Fig. 4, where 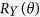 is defined as 
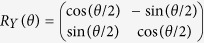
 in the computational basis. In Fig. 3a, 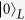 is first prepared in a non-fault-tolerant manner. Next, the encoded Hadamard operator is measured fault-tolerantly. The fault-tolerant measurement consists of fault-tolerant preparation of a seven-qubit cat state followed by transversal controlled-Hadamard gates and the measurements of the ancilla qubits. If the parity of the measurement results is even, then the measured encoded state is projected into 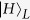, and otherwise projected into the state orthogonal to 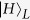, which is denoted by 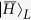. The fault-tolerant measurement is followed by error detection, which ensures that the system is in the code space. The whole procedure without the preparation of the logical zero state is repeated twice. If no error is detected and the two results of the fault-tolerant measurements are equal, then 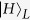 or 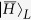 is prepared fault-tolerantly. Otherwise, the output state is rejected and the state preparation is restarted. In the case of 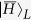, we can obtain 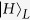 by applying transversal *Y* gates.

Figure 3b shows the proposed method for fault-tolerant preparation of 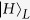. This method is also based on the fault-tolerant measurement of the encoded Hadamard operator. The differences from the conventional method described above are the following two points. First, the initial state is 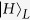, not 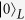, as in the case of magic state distillation^11^,^17^,^19^,^20^,^21^,^22^. This change allows one to remove the two-time repetition of the whole procedure in the conventional method. Second, the ancilla for the fault-tolerant measurement of the encoded Hadamard operator is a Bell state, not the seven-qubit cat state. This change becomes possible by *X*-error detection at the final measurement *M*_0/1_ for the ancilla. This technique was proposed in ref. 17 for step-by-step magic state encoding for concatenated quantum codes.

To confirm that the present method works successfully, we performed numerical simulation of a logical 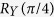 with 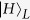. Here note that the stabilizer simulator mentioned above cannot be used for the present purpose, because the magic state is a nonstabilizer state. The present simulation is performed with a fully quantum-mechanical simulator devised by us for this work. The error model assumed here is the same as the above one.

**Figure 5:**
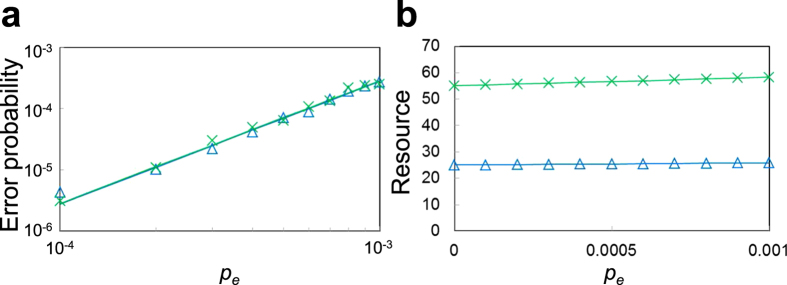
Simulation results of a logical *R*_*Y*_(*π*/4) with  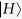. (**a**) Log-log plot of error probabilities of a logical 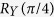. The straight lines are the fits of 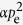 to the plots, where *α* is a fitting parameter. (**b**) Linear plot of resources of a logical 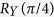, where the resource is defined as the total number of physical qubits. In both (**a**) and (**b**), the crosses and the triangles correspond to the cases where 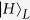 is prepared by the methods shown in Fig. 3a,b, respectively. *p*_*e*_ denotes the error probability per physical two-qubit gate.

From the simulation results, we estimated the error probability and total number of physical qubits for a logical 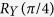, which are shown in Fig. 5a,b, respectively. In Fig. 5, the crosses and the triangles correspond to the cases where 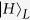 is prepared by the methods shown in Fig. 3a,b, respectively. (See Methods for the details of the simulation.)

## Discussion

First, we discuss the simulation results for a logical CNOT gate shown in Fig. 2. Since a logical CNOT gate requires two Bell states for error-correcting teleportation and each Bell state requires two 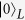, the resource for a logical CNOT gate is four times as many as the preparation of 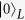. This is confirmed from Fig. 1 and Fig. 2b. The resources in Fig. 2b increase a little as *p*_*e*_ increases. This comes from the repetition due to postselection based on error detection. As is obvious from Figs 1 and 2b, the present method can achieve the fewest resources.

Figure 2a shows that all the methods can achieve the fault tolerance, because all the plots are well fitted with 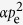, where *α* is a fitting parameter. Figure 2a also shows that the present method can achieve a logical error probability as low as the conventional ones. Thus, it turns out that the present method realizes the fault-tolerant preparation of 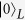 with fewer resources without increasing the logical error probability.

Next, we discuss the simulation results for a logical 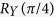 with 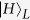 shown in Fig. 5. Since the error detection in Fig. 3 requires a Bell state for error-detecting teleportation and the Bell state requires two 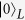, the resource for 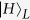 is the number of physical qubits shown in Fig. 3 plus 16 for two 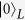. This is confirmed from Fig. 3 and Fig. 5b. (For the conventional method shown in Fig. 3a, the two-time repetition must be considered.) The resources in Fig. 5b increase a little as *p*_*e*_ increases. This comes, again, from the repetition due to postselection based on error detection. The present method can achieve fewer resources than the conventional one, as expected.

Figure 5a shows that both the methods can achieve the fault tolerance, because the plots are well fitted with 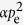, where *α* is a fitting parameter. Figure 5a also shows that the logical error probability for the present method is very close to that for the conventional one. Thus, it turns out that the present method realizes the fault-tolerant preparation of 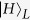 with fewer resources without increasing the logical error probability.

In conclusion, we have proposed efficient fault-tolerant preparation methods for zero and magic states encoded with the Steane code. By numerical simulations, it has been shown that both the methods can achieve fault tolerance with significantly fewer resource overheads without increasing logical error probabilities, compared to conventional methods. The overhead for the encoded zero state is only one physical ancilla qubit. This has become possible by careful consideration of error degeneracy, which is a nonclassical property of quantum codes. This result suggests that the nonclassical feature of the error degeneracy will be useful for improvement of fault-tolerant quantum computation. On the other hand, the overhead for the encoded magic state is only two physical ancilla qubits together with additional 16 physical qubits for error detection. It seems difficult to realize further reduction of the overhead. Thus, the present methods minimize the resource overheads for fault-tolerant state preparation with the Steane code.

## Methods

### The Steane code

The Steane code is a [[7,1,3]] stabilizer code, where [[*n*,*k*,*d*]] means that *k* qubits are encoded into *n* qubits and the code distance is *d*. The “check operators” (stabilizer generators) are defined by *IIIZZZZ*, *ZZIIZZI*, *ZIZIZIZ*, *IIIXXXX*, *XXIIXXI*, and *XIXIXIX*, which are based on the [[7,4,3]] classical Hamming code. The decoding algorithm for the measurement results of the check operators is the same as that for the Hamming code. The Steane code enables to correct arbitrary single-qubit errors and to detect arbitrary two-qubit errors in the code block, because the code distance is three. The encoded Pauli operators are defined by *XXXXXXX* and *ZZZZZZZ*. The encoded operators have different representations equivalent to one another because of the stabilizer operators. For instance, *XXXXXXX* is equivalent to *XXXIIII*. For the Steane code, all the Clifford gates can be implemented transversally.

### Simulation methods

The simulation method for a logical CNOT gate is the same as that in ref. 17. In this simulation, the logical CNOT gate is performed repeatedly ten times on the first logical qubits of two error-free logical Bell states. After that, the Bell states are disentangled by error-free operations. Finally, the logical qubits are measured to check whether logical errors have occurred or not. The error probabilities and resources are estimated by dividing those of the simulation results by ten. The simulator used here is the stabilizer simulator used in refs 16 and 17, which is based on ref. 18.

In the simulation of a logical 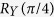 with 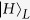, 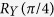 is performed repeatedly 16 times on the first logical qubit of an error-free logical Bell state. Note that the logical Bell state returns to the initial state after the operations. Here in the preparation of 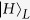 shown in Fig. 3, the error detection is implemented by error-detecting teleportation^7^,^16^, where the Bell state for this is prepared with 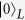 prepared by the method shown in Fig. 1c. Finally, the Bell state is disentangled by error-free operations and measured to check whether logical errors have occurred or not. The error probabilities and resources are estimated by dividing those of the simulation results by 16. The simulator used here is a fully quantum-mechanical simulator devised by us for this work.

## Additional Information

**How to cite this article**: Goto, H. Minimizing resource overheads for fault-tolerant preparation of encoded states of the Steane code. *Sci. Rep.* **6**, 19578; doi: 10.1038/srep19578 (2016).
