# Publisher Correction: Minimizing resource overheads for fault-tolerant preparation of encoded states of the Steane code

**DOI:** 10.1038/s41598-022-06633-6

**Published:** 2022-02-09

**Authors:** Hayato Goto

**Affiliations:** Frontier Research Laboratory, Corporate Research & Development Center, Toshiba Corporation, 1, Komukai Toshiba-cho, Saiwai-ku, Kawasaki-shi, 212-8582 Japan

Correction to: *Scientific Reports* https://doi.org/10.1038/srep19578, published online 27 January 2016

The original PDF of this article contains an error in the footer, where the volume number "5:19578" should read "6:19578".

